# Characterising contaminants distribution in marine-coastal sediments through multivariate and nonparametric statistical analyses: a complementary strategy supporting environmental monitoring and control

**DOI:** 10.1007/s10661-022-10617-4

**Published:** 2022-11-03

**Authors:** Alberto Ferraro, Alessandro Parisi, Enrico Barbone, Marco Race, Matilda Mali, Danilo Spasiano, Umberto Fratino

**Affiliations:** 1 Department of Civil, Environmental, Land, Building Engineering and Chemistry, Polytechnic University of Bari, Via E. Orabona 4, Bari, 70125 Italy; 2 Environmental Protection and Prevention Agency of Puglia Region (ARPA Puglia), Corso Trieste 27, Bari, 70126 Italy; 3 Department of Civil and Mechanical Engineering, University of Cassino and Southern Lazio, Via di Biasio 43, Cassino, 03043 Italy

**Keywords:** Marine sediments, Coastal waters, Environmental monitoring, Cluster analysis, Aquatic biota, Water Framework Directive

## Abstract

This work investigates a statistical approach analysing data from monitoring activities on marine-coastal areas for environmental quality determination and surveillance. Analyses were performed on a database of the Environmental Protection and Prevention Agency of the Puglia Region. As, Cr, Ni, and Pb concentration values in marine sediments and biota from 2013 to 2015 and 2017 were processed to investigate different contaminant characteristics. Hierarchical cluster analysis identified three contaminant distribution classes with (1) highest Cr, Ni, and Pb concentrations, (2) highest As concentration, and (3) lowest contaminants concentration. The Kruskal-Wallis and Friedman tests showed that contaminant distributions were statistically different when considering the monitoring years and classes. However, statistical similarities resulted during the 2013–2017 and 2014–2015 periods. Spearman’s coefficients displayed positive correlations among the pollutants in each matrix and mainly negative correlations for matrices comparison. This methodology aims to provide a practical support for monitoring to identify potential environmental deterioration over time and correlations with specific contamination sources.

**Supplementary Information:**

The online version contains supplementary material available at 10.1007/s10661-022-10617-4.

## Introduction

Marine water and sediment contamination is recognised as one of the most serious threats to environmental quality and human health. The continuous development of anthropogenic activities (agricultural, urban, and industrial) leads to a concurrent increasing pressure and consequent contaminant diffusion in the marine environment which is characterised by a significant vulnerability (Naifar et al., 2018). For instance, potentially toxic elements (PTEs) have a notable tendency to accumulate on marine sediments through adsorption phenomena to an extent depending on various factors (e.g. sediments texture and particle size, mineralogical composition) (Billah et al., 2017; Buccolieri et al., 2006). Moreover, the concentration trend of different PTEs (such as As, Ni, Cr, and Zn) can be considered an important indicator of the contamination source in the marine environment due to their strict correlation with the type of anthropogenic activity in a specific area (Burton et al., 2004).

In accordance with Water Framework Directive 2000/60/EC and Marine Strategy Framework Directive 2008/56/EC, continuous monitoring of different marine ecosystem descriptors was required to assess their quality and environmental status. Then, monitoring programmes (operational or surveillance) and survey technologies were implemented to properly evaluate the environmental quality of the marine environment (Bean et al., 2017; Beiras, 2018; Danovaro et al., 2016). In general, water quality monitoring involves deepened analysis on physical–chemical and biological parameters as well as contaminants detection. However, it has been demonstrated that sediments, compared to marine water, can represent a more suitable matrix to perform long-term studies useful for potential contamination detection (Ausili et al., 2020; Mali et al., 2018, 2019; Tavakoly Sany et al., 2014). Also, aquatic biota can further provide important information about marine contamination occurrence due to its specific characteristics in pollutants bioaccumulation (Gao et al., 2021).

As regards the contamination from PTEs, different studies reporting statistical analysis on data from marine environment monitoring can be found in previous literature. For instance, Pearson’s correlation analysis was generally used to assess the potential correlation between PTEs in marine sediments and environmental factors (Bastami et al., 2015; Liu et al., 2015). Application of hierarchical cluster analysis (HCA) to classify different sampling sites in accordance with the sediment quality guidelines (SQG) was reported for a case study on marine sediments (Mali et al., 2017, 2020; Zhuang & Gao, 2014). Multivariate statistical methods were also used to identify correlation between contaminants concentration in marine sediments and wave hydrodynamics conditions of the monitored area as well as the potential source of the contamination (Giglioli et al., 2020; Zhou et al., 2007).

The above-mentioned statistical methods highlight the efficacy and usefulness of data analysis applied during routine monitoring of marine water and sediments for the identification of potential sources of contamination on a larger scale. However, many studies have focused on detailed monitoring activities carried out in areas characterised by assessed or historical forms of contamination. Besides these cases, it is worth mentioning that data analysis of contaminants distribution in the marine environment could represent an overall fundamental tool for quality deterioration prediction and control in specific areas when carried out over long-lasting periods. This approach should be followed whether the contaminant concentrations are within or exceeding the threshold limits for environmental safety. Linked to this aspect, the identification of contaminant sources is further crucial to prevent the development of high pollutant concentration in marine-coastal waters.

The present work focused on the study and statistical elaboration of a database resulting from four campaigns of Operative Monitoring Plans carried out by the Environmental Protection and Prevention Agency of the Puglia Region (ARPA Puglia) on the whole coastal area of the region in south-eastern Italy. The main aim of the present work is to develop a practical statistical approach as a complementary tool for monitoring activities in the marine-coastal environment. This approach might provide a useful support in (i) contaminants distribution characterisation in specific areas of interest, (ii) identification of areas worthy of further detailed analysis/research, and (iii) recommendation of technical interventions aimed at preventing significant forms of contamination.

## Materials and methods

### Monitoring area

**Fig. 1:**
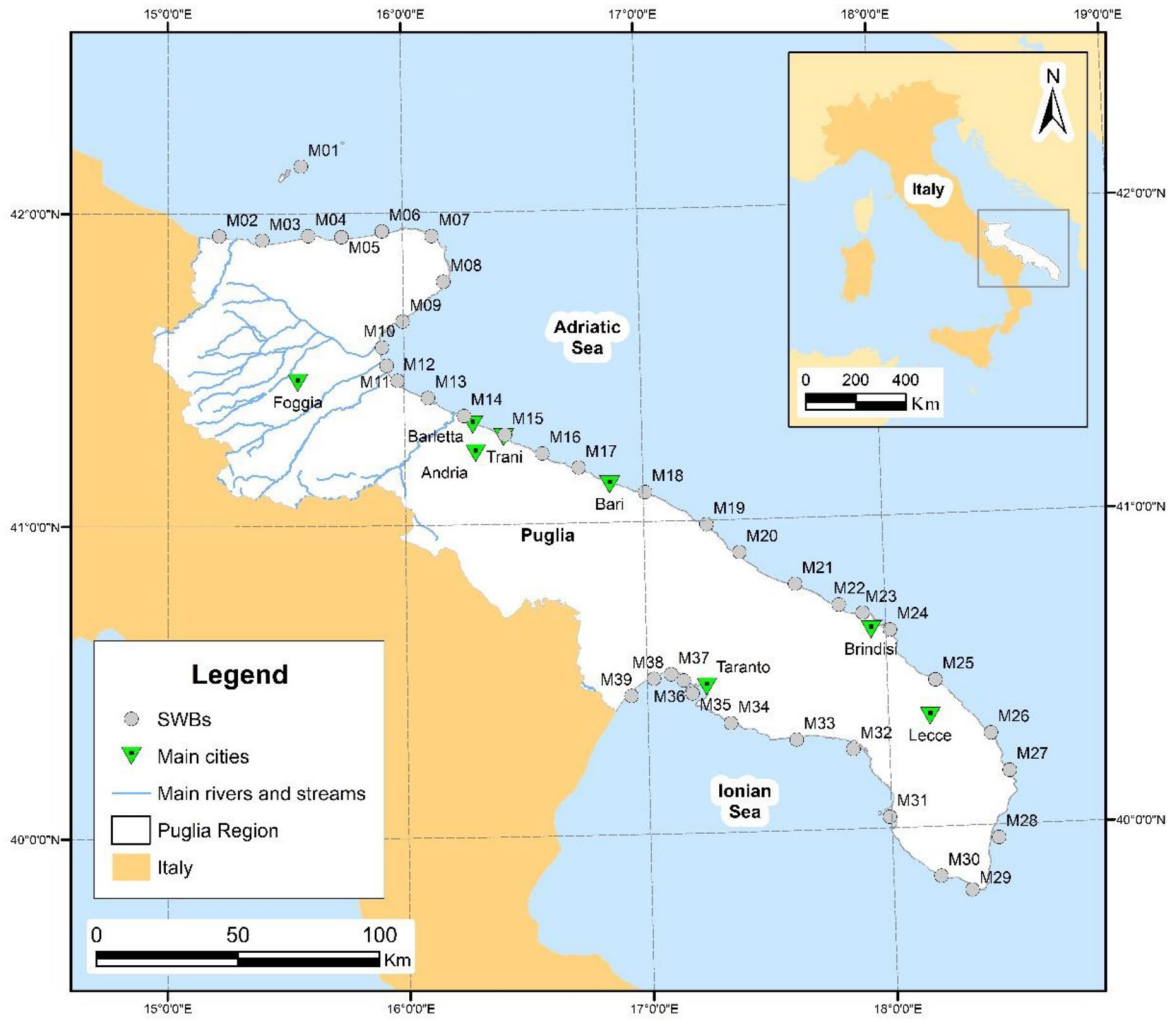
Framing of the Puglia Region and centroids of marine-coastal SWBs according to the Regional Water Protection Plan

The Puglia Region has a coastline of about 1000 km exposed to two seas (Fig. 1). The eastern part of the Puglia coastline and the Tremiti islands face the Adriatic Sea whereas the south-western coastline faces the Ionian Sea. As a consequence of its hydrological, geological, and geomorphological characteristics, the Puglia coastline is characterised by high rocky cliffs, river mouths, sandy beaches, and rocky coasts with a relevant tourist vocation (Malcangio et al., 2018). Water and sediment discharge towards the Adriatic and Ionian Seas is due to several inland sources. Among them, rivers and streams with the most significant flow, as well as some drainage water networks are mainly located in the north-eastern part of the region identified as the Tavoliere plain. Fossil and ephemeral streams with shore-orthogonal evolution typical of karst environments in semi-arid areas, as well as karst coastal springs discharging towards both the Adriatic and Ionian Seas, are mainly located in the Gargano, Murgia, and Salento areas, which are affected by extensive karst phenomena (Regional Water Protection Plan from http://www.sit.puglia.it/portal/portale_pianificazione_regionale/Piano%20di%20Tutella%20delle%20Acque/Documenti, last accessed on 16/09/2022). During the last decades, coastal areas experienced changes in land use towards a more intensive exploitation of inland natural resources, as well as a growing and high rate of urbanisation caused by urban development, transport infrastructure, harbours, and the presence of residential, tourist, commercial, and industrial activities (Bruno et al., 2021). Thus, periodical monitoring and control are essential to prevent unexpected and negative consequences on water bodies and water-dependent ecosystems.

### Database elaboration

With the aim of monitoring the quality of Puglia’s marine-coastal waters, sediments, and biota, according to the Italian Legislative Decree No. 152/2006 that implemented the Water Framework Directive 2000/60/EC, the Regional Water Protection Plan (from 2009 to 2015 and 2015 to 2021) defined, within a Surveillance Monitoring programme, 39 homogeneous marine-coastal surface water bodies (SWBs) based on hydrodynamic settings, as well as geomorphological characteristics (Table S1 of the Supplementary material). The Regional Water Protection Plan defined the position of at least two sampling points related to marine waters, sediments, and biota located in each marine-coastal SWB. Sampling points were identified on parallel transects orthogonal to the coastline, one within 500 m from the coastline and the other at the end of the nautical mile, which correspond to the regional administrative limit of marine-coastal SWBs. In the present work, data of PTE concentrations related to marine sediment and biota samples were provided by ARPA Puglia. This agency is responsible for the monitoring of regional marine-coastal waters, sediments, and biota in accordance with the Government Decree 116/2008 and with the Regional Water Protection Plan. In this paper, no PTE concentration in marine waters was considered due to the generally low concentration values detected.

For the purpose of the present work, data from 4 years corresponding to the Operative Monitoring Plan of 2013, 2014, 2015, and 2017 were considered (original database for 2013, 2014, and 2015 from https://www.arpa.puglia.it/pagina2976_i-ciclo-sessennale-2010-2015.html, last accessed on 16/09/2022; original database for 2017 from https://www.arpa.puglia.it/pagina2975_ii-ciclo-sessennale-2016-2021.html, last accessed on 16/09/2022). The latter decision was based upon the necessity to have an exact correspondence of sampling points among all monitoring years providing a homogeneous set of observations over time. Since all the marine-coastal SWBs had more than one sampling point, the averages of the concentration values of each PTE in the different sampling points of the investigated area were considered. Only 33 of 39 marine-coastal SWBs had a complete dataset related to PTE concentrations in sediment during the above-cited years. On the contrary, the 6 SWBs with incomplete PTE values over the whole monitoring period (i.e. M02, M03, M28, M29, M30, and M31) were not considered for further analyses. Accordingly, PTE concentrations with reference to 132 measures (33 homogeneous areas times 4 years) were considered eligible for the application of the following statistical analyses. Accounting for the biota, the same number of homogeneous areas compared to the sediments was only observed for 2013 and 2014. Instead, only 31 and 15 homogeneous marine-coastal areas equal for both marine sediments and biota were available for 2015 and 2017, respectively. As for the biota species, most of the organisms analysed for the evaluation of the bioaccumulation of contaminants during the monitoring activities considered in this study belong to the class of Holothuroidea.

Prior to statistical analyses, the original database of ARPA Puglia was further elaborated by replacing censored values for each variable (i.e. PTEs). To this aim, censored values replacement was carried out by multiplying all the “less than” data by a factor of 0.55 (Sanford et al., 1993). Moreover, all the variables which required more than 10% replacement were not further considered. The original database was composed of 7 PTEs (i.e. As, Cd, total Cr, Cr VI, Hg, Ni, and Pb) and 88 organic contaminants divided into 16 macro groups (i.e. organochlorine pesticides, chlorinated solvents, pentachlorophenol, alkylphenol, CCl_4_, organophosphate pesticides, PCBs, Di(2-ethylhexyl) phthalate, PAHs, organotin compounds, phytosanitary products, urea herbicides, aromatic solvents, PCB and dioxins, heavy hydrocarbons). Then, excluding the variables characterised by more than 10% of censored values replacement, a new database on marine-coastal sediments was obtained with four variables (As, total Cr, Ni, and Pb) associated to 33 homogeneous marine-coastal SWBs monitored over the 4 years (2013, 2014, 2015, and 2017). The same variables were also considered for the marine biota to properly determine potential correlations with the sediment matrix. Indeed, the selection of four PTEs for further database analysis would represent a hindrance for studies focusing on the assessment of a specific environmental contamination. On the contrary, it is worth highlighting that, in this case, the reduced number of variables did not represent a limitation since the aim of the present work was to develop a strategy for monitoring activities support instead of the determination of a contamination condition.

### Statistical analysis

#### Coastal area classification

Classification of the 33 monitored marine-coastal SWBs was carried out by considering the PTEs (As, Cr, Ni, and Pb) concentration detected in marine sediments. Initial PTE values were standardised prior to the HCA in order to avoid the influence of outliers on the final classification. Then, the HCA was performed by means of Ward’s linkage algorithm used in combination with the squared Euclidean distances as nearness criteria (Abu-Jamous et al., 2015). The optimal number of clusters was selected through the elbow method which considers the total variance within the different clusters and higher cluster uniformity is obtained for lower variance values (Crowther et al., 2021).

In order to provide further insights, the HCA results and land use of coastal areas were compared. Land use data with 8 m resolution and updated to 2011 was provided by the Puglia Region which followed the nomenclature of Corine Land Cover classes (data from http://www.sit.puglia.it/portal/portale_cartografie_tecniche_tematiche/Cartografie%20Tematiche/UDS, last accessed on 16/09/2022). Land use analysis was carried out for inland coastal basins associated to homogeneous marine-coastal SWBs in accordance with the Regional Water Protection Plan. Each inland basin extends up to 3 km from the coastline. Land use analysis considering this inland basin extension made it possible to identify the main influencing activities close to the marine-coastal areas. As successively described, percentages of land uses were calculated considering contiguous inland basins where marine-coastal SWBs resulted in the same HCA class for all the monitoring periods.

#### Nonparametric analyses on marine sediments and biota contamination

Due to the non-normal distribution of the data investigated in this work, statistical analyses aimed at providing further understanding of the presence of contaminants in the marine-coastal sediments were performed through nonparametric tests. For the latter, despite the limited information achievable compared to parametric approaches, the main advantages are represented by the unnecessary hypothesis assumption on data distribution and the lower influence of potential outliers on the analysis outcome (Nahm, 2016). A first nonparametric analysis was carried out through the Friedman statistical test to identify if significant differences were observable among variables during the monitoring years. In order to determine the monitoring years leading to the null hypothesis (H_0_) rejection, the Nemenyi post hoc test was used for variables pairwise comparison after the Friedman test (Verma & Ranga, 2020).

Furthermore, the Kruskal-Wallis test was performed, for each year, to highlight potential differences among the marine-coastal SWBs (considered as a function of the related HCA class) in terms of distribution of contaminants. Also in this case, a post hoc test, namely the Dunn multiple-comparison test, was carried out to investigate classes displaying more significant differences compared to the others. All the analyses were performed at a 5% significance level. Moreover, a Bonferroni correction of the *p*-values was performed for the Dunn multiple-comparison test to reduce the type I error probability (Bai et al., 2020).

A nonparametric Spearman test was carried out to determine the correlation coefficients among marine-coastal sediment contaminants related to each specific HCA class over the whole monitoring period. Similarly, potential correlations were provided for the same contaminants but in the biota matrix as well as for the overall comparison in both marine sediments and aquatic biota. Also in these cases, the determination of the Spearman correlation coefficients was performed considering the data for sediments and biota over the whole monitoring period but still discriminating the contaminants values by HCA classes. The level of significance of the statistical correlations was set equal to 5%.

## Results

### Determination of contaminant distribution classes through HCA

According to the results from the HCA and the total variance determination through the elbow method, the optimal number of classes was set equal to 3 (identified as C-1, C-2, and C-3). In detail, the classes denoted three different contaminant distributions corresponding to (i) the overall highest Cr, Ni, and Pb concentrations and average As concentration for C-1, (ii) the overall highest As concentration and average Cr, Ni, and Pb concentrations for C-2, and (iii) the overall lowest contaminants concentration for C-3.

**Fig. 2:**
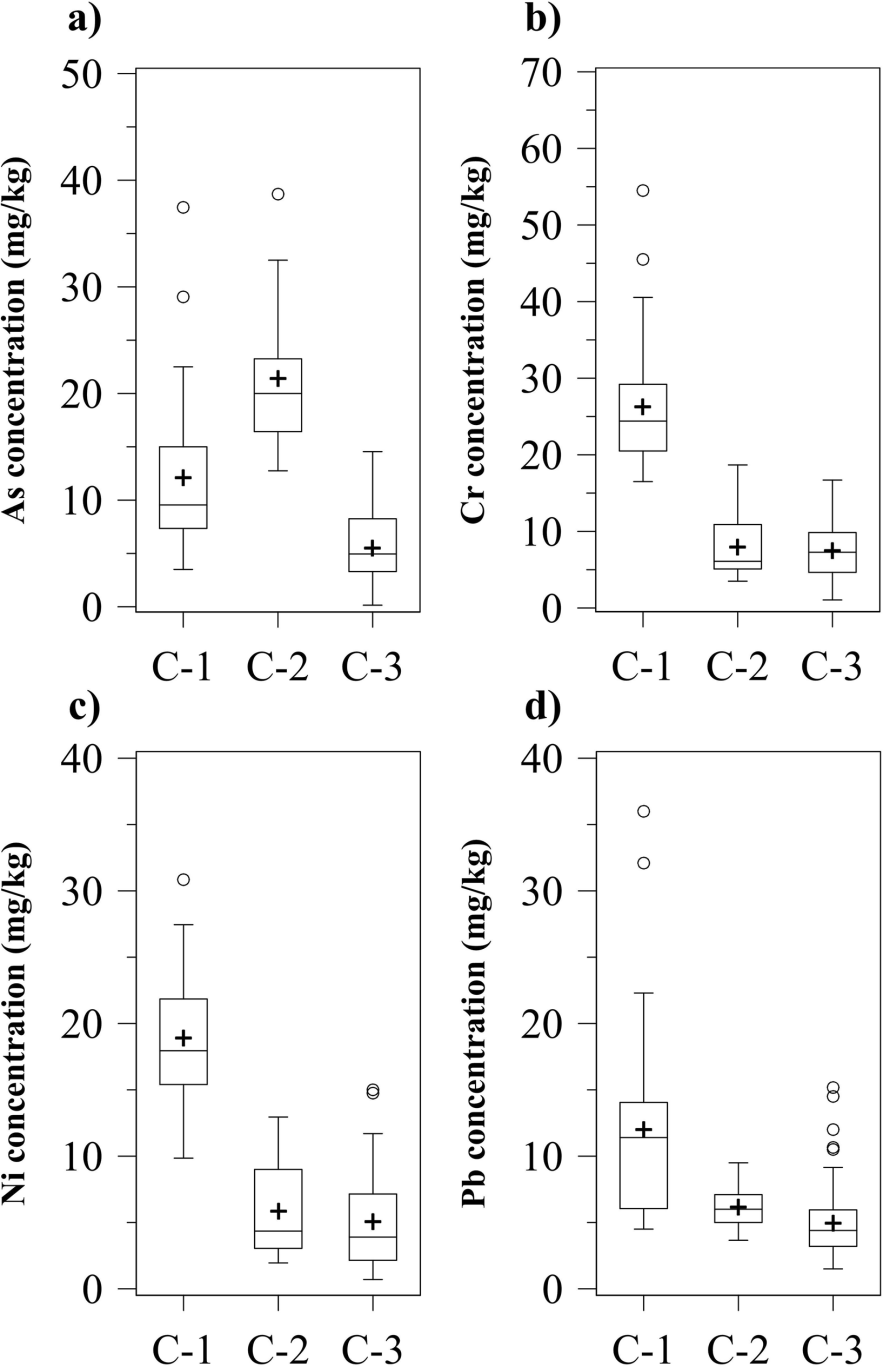
Box plots of the **a** As, **b** Cr, **c** Ni, and **d** Pb concentration profiles in marine sediments for each class over the whole period of investigation. For each plot, mean concentration values (black crosses), outlier concentration values (white dots), standard deviations (bars), sample median, first quartile (25th percentile), and third quartile (75th percentile) were reported

Contaminant concentration profiles in the marine sediments for each class can be observed in Fig. 2. Overall contaminant mean concentrations of C-2 were slightly higher compared to C-3 (Fig. 2b, c, and d). In particular, mean concentration values for Cr, Ni, and Pb were respectively equal to 7.95, 5.88, and 6.20 mg/kg in C-2 and 7.48, 5.05, and 4.99 mg/kg in C-3. On the contrary, Cr, Ni, and Pb concentrations of C-1 were significantly higher compared to C-2 and C-3. Also, despite a lower As mean concentration compared to C-2 (12.10 vs. 21.41 mg/kg for C-1 and C-2, respectively), it was possible to observe similar As maximum concentration values achieved in both classes (37.45 vs. 38.70 mg/kg for C-1 and C-2, respectively) (Fig. 2a). Further maximum concentration values resulting in C-1 were equal to 54.50, 30.85, and 36 mg/kg for Cr, Ni, and Pb respectively. It is worth noticing that few outlier values were generally observed for all the investigated contaminants.

**Fig. 3:**
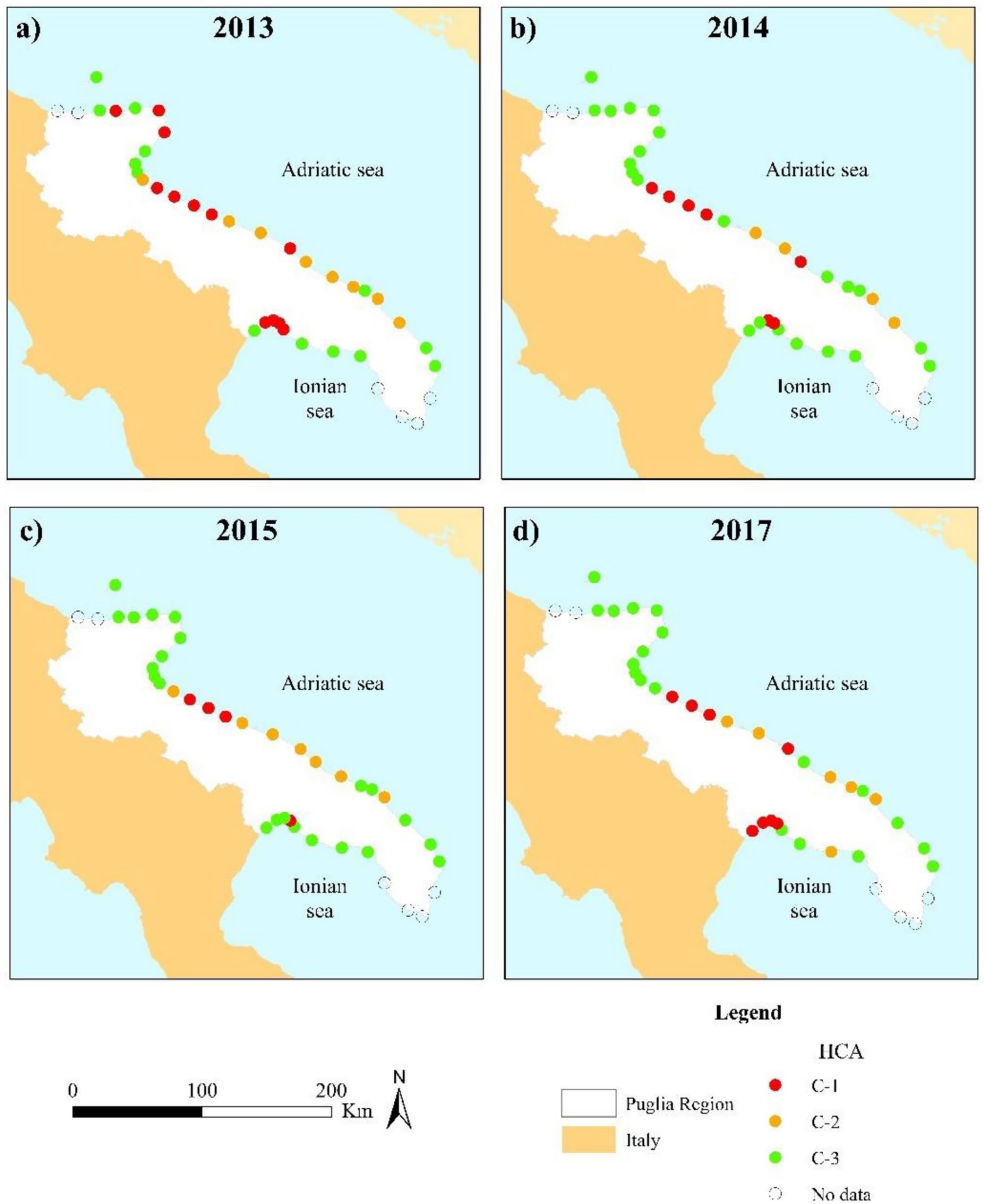
Maps of the HCA classes spatial distributions during **a** 2013, **b** 2014, **c** 2015, and **d** 2017

Figure 3 shows the results of HCA displaying a variable trend in terms of classes spatial distribution among the monitoring years. In general, higher presence of areas within C-1 and C-2 was observed during 2013 (Fig. 3a). In this year, the two classes were mainly observed on the Adriatic coastal areas with C-1 prevalence in the north-eastern part of the Puglia Region and a noticeable presence also on the Ionian coastal side. On the contrary, areas classified in C-2 were all related to the Adriatic Sea. As can be observed, an overall improvement occurred in 2014 (Fig. 3b) with the presence of C-3 areas increasing especially in the north-eastern Adriatic areas and the Ionian side which displayed fewer areas with high concentrations of PTEs also in 2015 (Fig. 3c). In particular, the 2015 monitoring year was characterised by the lowest presence of homogeneous areas classified as C-1 and by the increase of C-2 areas becoming prevalent in the mid zone of the Adriatic Sea. Finally, spatial distribution of 2017 (Fig. 3d) further highlighted a new increase of area classified as C-1 on both the Adriatic and Ionian sides. In particular, the latter was indeed characterised by the most significant increase of C-1 areas compared to 2015. Moreover, the first C-2 area on the Ionian coast was observed in 2017.

It is worth highlighting that, also for the C-1 class, contaminant concentration values were not always higher than thresholds identified with the standard environmental quality mean annual values (SQA-MA) reported by the Italian Regulation (Legislative Decree n. 172/2015). In particular, total Cr concentration slightly higher than the related SQA-MA (i.e. 50 mg/kg) was observed in one C-1 area during 2017. Similarly, slightly higher Pb values than SQA-MA (i.e. 30 mg/kg) were observed for two C-1 areas each detected in different years (2013 and 2017). On the contrary, As values higher than the SQA-MA threshold (i.e. 12 mg/kg) resulted for more areas irrespective of the class or the monitoring year. In detail, nine C-1 areas in the 4 years were characterised by values ranging from 12.4 to 37.5 mg/kg and two C-3 areas with concentrations of 13.1 and 14.6 mg/kg were observed in 2015 and 2017, respectively. As expected, all the C-2 class areas displayed As values higher than SQA-MA from 2013 to 2017.

### Nonparametric statistical analyses

Descriptive statistics from the Kruskal-Wallis test performed on marine sediments contaminants in order to evaluate significative differences among classes (C-1, C-2, and C-3) for each monitoring year are reported in Table S2 of the Supplementary material.

According to the statistical results, it was possible to notice statistically significant differences (*p* < 0.05) during the monitoring years for all the contaminants highlighting their different data distributions when compared among classes. However, statistical differences and similarities among classes were different according to the contaminant considered. This aspect was further stressed by the outcomes from the post hoc Dunn test (Table 1).

**Table 1:** Dunn post hoc test results on similarities/differences for each contaminant among the HCA classes respective to the monitoring year

**Year**	**As**			**Cr**			**Ni**			**Pb**		
	**C-1**	**C-2**	**C-3**	**C-1**	**C-2**	**C-3**	**C-1**	**C-2**	**C-3**	**C-1**	**C-2**	**C-3**
**2013**	B	B	A	B	A	A	B	A	A	B	A, B	A
**2014**	D	D	C	D	C, D	C	D	C, D	C	D	C, D	C
**2015**	E, F	F	E	F	E	E	F	E, F	E	F	E, F	E
**2017**	G, H	H	G	H	G	G	H	G	G	H	G, H	G

Significant differences were observed for a Bonferroni corrected level of significance equal to 0.0167

In Table 1, for each contaminant and respective to the monitoring year, significant differences among the classes were denoted by association to different letters. The Dunn test results highlighted that quite similar differences could be observed for each contaminant among the classes. Accounting for the C-2 class, more similarities with only the C-1 class were displayed for As. However, during 2015 and 2017, As concentration in C-1 showed no statistical differences with any of the other classes. For the other contaminants, C-2 showed either predominant similarity with C-3 (such as for Cr) or almost no difference with any of the classes (such as for Ni and Pb). In general, except for the As in 2015 and 2017, the main significant differences were always observed between C-1 and C-3 as expected from the HCA classification results.

From Friedman’s test results (Table S3 of the Supplementary material), it was possible to observe that statistical differences of contaminant distributions among the monitoring years occurred for all the investigated PTEs except Pb which displayed a *p*-value higher than 0.05. According to this, the Nemenyi post hoc test was useful to identify the monitoring years that were mainly responsible for the statistical differences observed.

Results from Nemenyi post hoc are reported in Table 2. Also in this case, various differences were observed among the monitoring years as a function of the considered variable. Accounting for the As, the main differences were observed when comparing the 2014 monitoring year with 2013 and 2017. On the contrary, Cr referred to 2014 displayed no differences compared to 2013 and 2017 while these 2 years were significantly different from the Cr data in 2015. A more variable result could be observed for Ni with the identification of three groups of similarity. In this case, the only significant difference was observed between Ni data from 2013 and 2014. Also, despite no significant difference between 2015 and 2017, the Ni content related to the 2 years displayed different similarities as compared to 2013 and 2014. In fact, Ni data distributions of 2015 and 2017 were significantly different compared to 2013 and 2014, respectively. As expected, the lack of differences in Pb data distribution among the monitoring years was further confirmed by the Nemenyi post hoc test.

**Table 2:** Nemenyi post hoc test results on similarities/differences for each contaminant among the monitoring years

**Variable**	**2013**	**2014**	**2015**	**2017**
**As**	B	A	A, B	B
**Cr**	D	C, D	C	D
**Ni**	G	E	E, F	F, G
**Pb**	H	H	H	H

Significant differences were observed for *p* < 0.05

### Statistical correlations of the contaminants

#### Correlations for contaminants in the marine sediments and aquatic biota

All the Spearman correlation coefficients resulting from the comparison among the investigated contaminants in the marine sediments for each HCA class are reported in Table 3.

**Table 3:** Spearman correlation coefficients among contaminants in marine sediments as a function of HCA classes

**Variable**	**As_C-1**	**Cr_C-1**	**Ni_C-1**	**Pb_C-1**	**As_C-2**	**Cr_C-2**	**Ni_C-2**	**Pb_C-2**	**As_C-3**	**Cr_C-3**	**Ni_C-3**	**Pb_C-3**
**As_C-1**	**1**	**0.420**	**0.478**	**0.379**								
**Cr_C-1**	**0.420**	**1**	**0.582**	0.225								
**Ni_C-1**	**0.478**	**0.582**	**1**	**0.360**								
**Pb_C-1**	**0.379**	0.225	**0.360**	**1**								
**As_C-2**					**1**	0.212	**0.426**	0.198				
**Cr_C-2**					0.212	**1**	**0.561**	0.393				
**Ni_C-2**					**0.426**	**0.561**	**1**	**0.731**				
**Pb_C-2**					0.198	0.393	**0.731**	**1**				
**As_C-3**									**1**	**0.404**	**0.421**	0.076
**Cr_C-3**									**0.404**	**1**	**0.590**	**0.249**
**Ni_C-3**									**0.421**	**0.590**	**1**	**0.318**
**Pb_C-3**									0.076	**0.249**	**0.318**	**1**

Bold values represent statistically significant correlation at *p* < 0.05

All the classes displayed positive correlations among the contaminants and most values were characterised by statistically significant correlations (*p* < 0.05). Among the Spearman correlations performed on marine sediments (Table 3), the highest positive coefficient values for all the classes were observed between Ni and Cr (0.582, 0.561, and 0.590 for C-1, C-2, and C-3 respectively). Moreover, significantly lower correlation values were displayed between Ni and As (0.478, 0.426, and 0.421 for C-1, C-2, and C-3 respectively). Also, the overall highest correlation coefficient value (0.731) was observed for the C-2 class and was still related to Ni when compared to Pb.

Also, for the correlations of the contaminants only related to the biota, an overall positive correlation for each class was reported (Table 4). However, in this case, generally higher correlation coefficient values could be observed compared to the Spearman correlation results on the marine sediments. In fact, the lowest correlation coefficient values determined for the biota were equal to 0.409 (between As and Pb) for C-1, 0.423 (between As and Pb) for C-2, and 0.416 (between Ni and Pb) for C-3. For marine sediments on the other hand, the lowest values were equal to 0.360 (between Ni and Pb), 0.198 (between As and Pb), and 0.076 (between As and Pb) for C-1, C-2, and C-3 respectively. In general, the highest correlations in the biota were observed between Ni and Cr for C-1 (0.902) and C-3 (0.702) and between Pb and Cr for C-2 (0.787).

**Table 4:** Spearman correlation coefficients among contaminants in biota as a function of HCA classes

**Variable**	**As_C-1**	**Cr_C-1**	**Ni_C-1**	**Pb_C-1**	**As_C-2**	**Cr_C-2**	**Ni_C-2**	**Pb_C-2**	**As_C-3**	**Cr_C-3**	**Ni_C-3**	**Pb_C-3**
**As_C-1**	**1**	**0.654**	**0.654**	**0.409**								
**Cr_C-1**	**0.654**	**1**	**0.902**	**0.712**								
**Ni_C-1**	**0.654**	**0.902**	**1**	**0.657**								
**Pb_C-1**	**0.409**	**0.712**	**0.657**	**1**								
**As_C-2**					**1**	**0.670**	**0.547**	0.423				
**Cr_C-2**					**0.670**	**1**	**0.773**	**0.787**				
**Ni_C-2**					**0.547**	**0.773**	**1**	**0.621**				
**Pb_C-2**					0.423	**0.787**	**0.621**	**1**				
**As_C-3**									**1**	**0.653**	**0.439**	**0.472**
**Cr_C-3**									**0.653**	**1**	**0.702**	**0.614**
**Ni_C-3**									**0.439**	**0.702**	**1**	**0.416**
**Pb_C-3**									**0.472**	**0.614**	**0.416**	**1**

Bold values represent statistically significant correlation at *p* < 0.05

#### Correlations between contaminants in marine sediments and aquatic biota

**Fig. 4:**
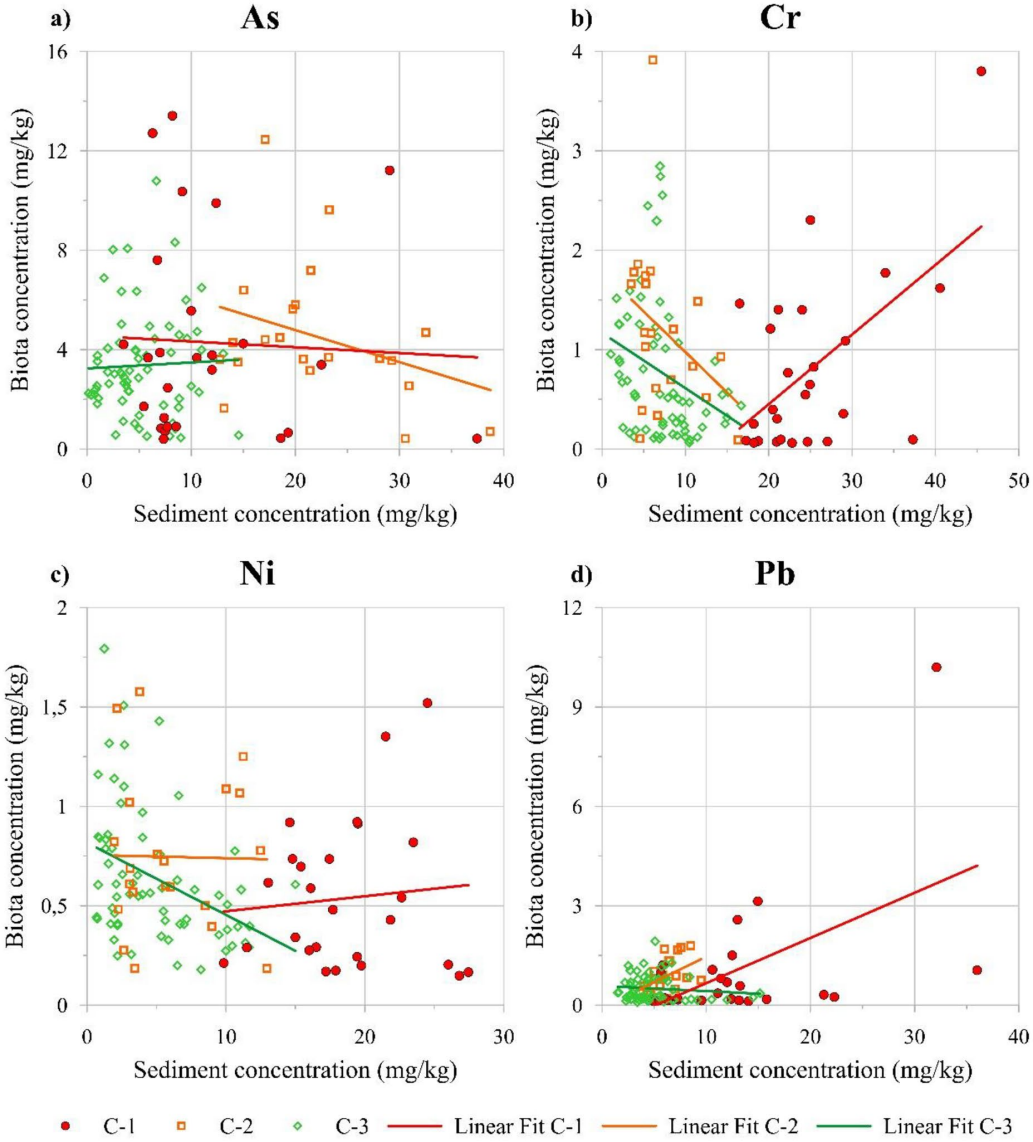
Scatter plots reporting the concentration trends of **a** As, **b** Cr, **c** Ni, and **d** Pb resulting from comparison between marine sediments and biota

From a preliminary comparison of each contaminant concentration in both marine sediments and biota, different trends were observed depending on the HCA class. Scatter plots in Fig. 4 represent the variation of each contaminant concentration in both environmental matrices for the three HCA classes. It is worth highlighting that a linear fit was reported in order to provide a general tendency of concentration profile for each contaminant in both matrices rather than a specific model describing the concentration variation. This was also noticeable from the results of the Spearman correlation for each PTE in both matrices (Table S4 of the Supplementary material) displaying few coefficients with statistically significant values at *p* < 0.05 (i.e. Cr in the C-2 and C-3 classes as well as Ni in the C-3 class).

Accounting for the C-1 class, increasing contaminant concentration profiles were observed in sediments and biota for all the contaminants with the exception of As (Fig. 4a). This suggested that increasing concentrations of Cr, Ni, and Pb in sediments could be related to a corresponding increase in the biota. On the contrary, decreasing profiles were observed for the C-2 and C-3 classes for almost all the contaminants. In particular, the only increasing concentration profile for C-2 was related to Pb (Fig. 4d) while a slightly decreasing one was shown for Ni (Fig. 4c). As for the C-3 class, decreasing concentration profiles were observed for all the contaminants except for As, which displayed an almost constant one. Finally, in the sole case of Cr (Fig. 4b), the concentration profiles of the three classes were markedly increasing for C-1 and decreasing for both C-2 and C-3.

Referring to the correlation among all the contaminants for both matrices, several negative coefficients could be observed in each class (Table S4 of the Supplementary material). In detail, the more statistically significant correlations for C-1 were both positive and negative. Weak positive correlations were displayed between Cr in sediments and Ni in the biota (0.396) as well as between Pb in sediments and Cr in the biota (0.462). A statistically significant negative correlation was instead observed between Ni in sediments and As in the biota (−0.551). Accounting for C-2, only one statistically significant positive correlation was observed between Pb in both sediments and biota (0.459). Finally, all statistically significant negative correlations were observed for C-3 with values ranging from −0.440 (between Ni in both sediments and biota) to −0.274 (between Ni in sediments and As in biota).

### Land use analysis

**Fig. 5:**
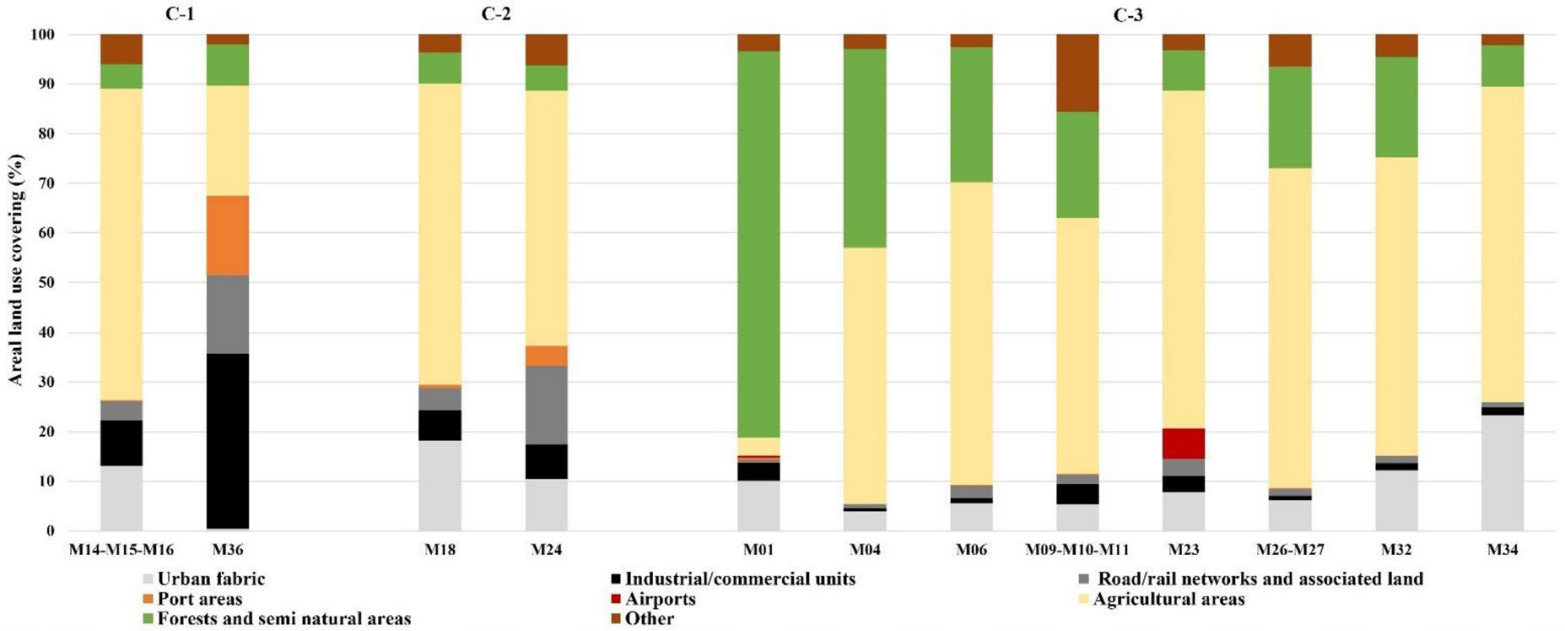
Percentage values of the areal land use covering for areas with same HCA classification during all the monitoring years

From the HCA results, the number of homogeneous marine-coastal SWBs in the same class for all the monitoring period was equal to 4 for C-1 (M14, M15, M16, and M36), 2 for C-2 (M18 and M24), and 11 for C-3 (M01, M04, M06, M09, M10, M11, M23, M26, M27, M32, and M34) (Table S1 of the Supplementary material). In general, agricultural areas land use displayed the highest areal percentage values for all the above-mentioned coastal-marine areas (Fig. 5). However, the main differences could be noticed when comparing the overall artificial surfaces (urban fabric, industrial, commercial, and transport units) identified in the first level of the Corine Land Cover classes. In fact, the overall artificial surface covering for C-1 and C-2 was always higher than C-3 with a range from 31.81 to 68.59% (Fig. 5). In the specific case of the M36 area (Punta Rondinella – Tara River mouth), the industrial-commercial-transport land use (industrial or commercial units, road and rail networks, airports, and port areas), identified at the second classification level of the Corine Land Cover classes, was significantly predominant (67.06%) compared to other land uses.

C-3 areas generally displayed an artificial surface covering ranging from 5.39 to 17.23%. Only for M23 (southern limit of Torre Guaceto protected marine area – Brindisi) and M34 (Torre dell’Ovo – Capo San Vito) these values were equal to 22.34 and 27.82%, respectively. However, M34 was characterised by only 2.63% of areas specifically devoted to industrial-commercial-transport activities. On the contrary, 12.79% of industrial-commercial-transport land use was observed for M23. The latter value was comparable to the same land use of the contiguous M14-M15-M16 areas (13.37%) for C-1 and M18 (11.28%) for C-2. Nonetheless, by further detailing the land use covering at the third classification level of the Corine Land Cover classes, the value reported for M23 was more ascribable to airports (6.12%) than to commercial and industrial settlements (3.30%).

## Discussion

The quality level of an environmental matrix can represent a fundamental indicator for possible consequences on human health as well as a parameter that is useful for providing important information about anthropic pressure and natural sources of contamination (Cioffi et al., 2021). For this purpose, a deep investigation of the potential aspects related to the contaminant distribution and corresponding variation over time is necessary. Accordingly, the present work focused on a monitoring strategy supported by statistical analysis to identify both contaminant sources and trends in the marine environment.

From the area classification provided by the HCA on data collected from 4 years of monitoring activities on marine sediments, it was possible to identify three specific contaminant distribution profiles. Indeed, based on characteristics of the identified classes, the quality standard observed could be considered in a range from “worst case” for the C-1 class to “best case” for the C-3 class. In general, the number of areas for each class was different during the monitoring years, identifying variable environmental quality conditions. Among the four investigated years, the highest presence of C-1 areas was observed during 2013, indicating an overall worst level of contaminant presence along the marine-coastal areas. Despite this, also during 2013, several PTE concentration values were lower or slightly higher than the SQA-MA thresholds. On the contrary, the significant increase of areas classified as C-3 during 2014 and 2015 highlighted better environmental conditions than 2013, while slight worsening could be still observed in 2017. This trend was consistent with results from the Nemenyi post hoc test which displayed the main similarities between the 2-year periods 2013–2017 and 2014–2015.

As regards the investigated contaminants, according to the observations from the HCA results and SQA-MA values, particular warning should be focused on As concentration due to the high concentrations reported. For Pb and total Cr, instead, monitoring activity could be important to investigate changes of concentration trends over time preventing further environmental deterioration. Accounting for the Ni concentrations, no SQA-MA value is regulated by the Legislative Decree n. 172/2015.

Despite this, positive correlations among contaminants higher than 0.5 were usually displayed when referring to Ni with Cr for C-1, C-2, and C-3 and Pb for C-2. This suggests that possible contamination sources should be referred to natural and/or anthropogenic phenomena/activities which are generally linked to the co-presence of Ni with Cr and Pb, respectively (Traven et al., 2015). In general, the above-mentioned PTEs can be concurrently present in marine sediments of marine-coastal areas characterised by significant anthropic pressure. In particular, this occurrence was previously observed for possible correlation between Ni and Cr due to industrial and shipyard activities (Baysal & Akman, 2018). Similarly, the co-presence of Ni and Pb in marine sediments was potentially linked to petroleum derivatives and antifouling paints related to shipyard activities (Pereira et al., 2018).

Accounting for the present work, results of the areal covering percentages, based on regional land use information, indicated a prevalence of industrial activities in areas classified as C-1 and C-2 during all of the monitoring period. Indeed, the three contiguous C-1 areas on the Adriatic Sea (M14-M15-M16) and the C-1 area on the Ionian Sea (M36) display different characteristics in terms of anthropic pressure on the marine-coastal environment. Specifically, a significant industrialisation is evidently present in the M36 area while an almost two-times lower industrial areal percentage was determined for M14-M15-M16. The latter areas are located in the Gulf of Manfredonia and are close to the mouth of the Ofanto River which is the major watercourse leading to the Southern Adriatic Sea (Mali et al., 2017). Besides high nutrients supply from agricultural areas to the coastline through freshwater transport possibly leading to eutrophication phenomena in the marine environment (Barbone et al., 2014), inland contribution can also represent a source of PTEs which may potentially reach high concentrations. In this area, there may also be a possible contribution deriving from inland sediments transported by the Ofanto and further mixed with northern marine sediments due to anticyclonic gyre occurrence in the Gulf central area (Fossile et al., 2021; Mali et al., 2019). Indeed, current circulation in the Adriatic Sea could effectively represent a further source of contaminated sediments in this area. More in detail, the anticyclonic circulation in the Southern Adriatic Sea may contribute to an additional marine sediments transport and deposition from the Albanian coast to the stretch of the Puglia coast in the southern part of the Gargano promontory. These sediments derive from the erosion of Albanian catchments containing mafic and ultramafic rocks and are characterised by high Ni and Cr content (Spagnoli et al., 2008). According to a previous study, a first transport is due to the Eastern Adriatic Current which leads to the accumulation of sediments highly contaminated by Ni and Cr in the Southern Adriatic Pit in front of the southern Croatian coast (Ilijanić et al., 2014). Then, the sediments are introduced in the anticyclonic circulation of the Southern Adriatic Sea. The above-reported discussion denotes the overall complexity in the comprehensive definition of a contaminant source and highlights the significance of a multidisciplinary monitoring strategy. However, it is worth noticing that contaminants retention in the marine sediment can be both temporally and spatially lasting. This provides useful information to track a contaminant source and further support the importance of marine sediments as a conservative environmental matrix for proper monitoring activities.

Also for the C-2 areas (M18 and M24), a high percentage of industrial activities is displayed along the coastline but the main contamination was characterised by As presence in the marine sediments. In specific cases, As can be linked to a natural background level for marine-coastal areas characterised by submarine volcanic sources (Giglioli et al., 2020). Nonetheless, As anthropic sources in the marine environment due to agricultural (pesticides, herbicides, etc.) and industrial activities are also reported (Baeyens et al., 2019). Accounting for the M18 and M24 areas, no presence of volcanic or thermal spring sources is indicated. Moreover, As correlation coefficients in the C-2 class did not display a particular link with other contaminants. This suggested that As presence in marine sediments of the C-2 class could be ascribable to industrial activities specifically related to this kind of contamination. Finally, low percentage values of the industrial area covering for C-3 clearly supported the results from the HCA analysis highlighting the lowest PTE concentrations in this class. However, also in this case, the effect of Adriatic current circulation could account for the slight PTEs presence in the marine sediments for some C-3 areas. For instance, regarding the northern Gargano promontory, the input of marine sediments discharged by the Po River and transported to the Puglia coast through the Western Adriatic Current can explain the detection of Ni and Cr (Lucchini et al., 2003). This is consistent with the Spearman coefficient between the two contaminants which displayed an average positive correlation for the C-3 class.

Positive Spearman coefficient values between contaminants in both marine sediments and biota did not display any prevalent correlation. Statistically significant positive correlations were always observed for the contaminants in the biota. This suggested a similar behaviour among the contaminants and no preferential accumulation by the marine organisms as a function of some specific PTE group. Moreover, few correlation coefficients with statistically significant values (*p* < 0.05) were observed for each PTE in both matrices while correlation coefficients among PTEs referring to each single matrix were characterised by numerous statistically significant values. Though the Spearman tests provided several correlation coefficients with statistically significant values assessing the results reliability, it is worth highlighting that some of these coefficients were characterised by weak or average values. This clearly highlights that not all the positive correlations among the investigated elements were strong and a more complex definition of contaminant behaviour can be expected when referring to different environmental matrices. Indeed, contaminant transfer from marine sediments to the biota can be extensively affected by several parameters as well as by the water phase influencing both release and accumulation phenomena. This suggests that more accurate studies should be carried out on the biota and its interdependence with environmental factors. Concurrently, further research activities should be devoted to the development of models for the assessment of the effects on contaminant concentration changes between matrices due to different environmental factors.

Despite this, accounting for the overall concentration profile of each contaminant in both matrices, it is possible to draw some preliminary considerations about the potential bioavailability of the PTEs investigated. The latter is an important parameter to consider for the assessment of contamination conditions in marine environments although its evaluation should take into account several influencing phenomena. Contaminant bioavailability can strongly influence the PTE bioaccumulation rate by marine organisms (such as fish and molluscs) leading to trophic transfer along the marine food chain (Zhang et al., 2016). Nonetheless, PTEs bioavailability in marine sediment can vary as a function of the environmental and redox conditions which could cause changes in the chemical speciation. Recent studies reported further effects on marine sediment properties due to bioturbation phenomena by organisms such as amphipods (Amato et al., 2016; Remaili et al., 2016). Indeed, an increasing bioturbation mix of sediment stratums entails the variation of redox conditions and consequent alteration of the binding strength between contaminant and solid matrix (de Jonge et al., 2012). Moreover, a further important parameter defining the binding strength of contaminants to marine sediments is represented by the fractionation of the pollutant within the solid matrix (i.e. contaminant distribution in exchangeable, reducible, oxidisable, and residual fractions).

According to the results on concentration profiles, slight Ni increase in the biota was observed for the C-1 class with increasing concentration in sediments. Instead, sharp contaminant increase in biota with increasing concentration in sediments was displayed for Cr in the C-1 class and Pb in the C-1 and C-2 classes, suggesting a higher bioavailability. This could be ascribable to the main presence of Cr and Pb in the more labile fractions of the marine sediments specifically identified as the exchangeable and reducible ones (Petranich et al., 2018). Considering this aspect, the need for proper monitoring activity that is also focused on these PTEs, despite the few concentration values exceeding the SQA-MA limits, is further evident. Moreover, the PTEs fractionation in marine sediments can provide further information about the contaminant source since high concentrations in more labile fractions are mainly associable to an anthropogenic origin (Gu et al., 2012). Besides this, opposite behaviour resulted for almost all PTEs in the C-3 class as well as for Cr and As in the C-2 class showing higher concentrations in biota at lower concentrations in sediments. A similar result was previously observed for the estuarine environment showing highest PTEs (such as As, Cd, Cu, Ni, and Pb) bioaccumulation in clams as well as cockles in an area characterised by lower sediment contamination (Figueira et al., 2011; Velez et al., 2015). This may be strictly related to the environmental characteristics of the investigated areas. For instance, high nutrients supply from agricultural areas can cause growth of phytoplankton which could represent a more significant PTEs source than sediment organic matter. A further example is provided by marine environments characterised by high Fe content which leads to increased contaminant bioavailability due to Fe complexation with organic matter (Figueira et al., 2011). Accordingly, attention should be paid also to marine areas characterised by low sediments contamination. This is fundamental to avoid potential risks due to contaminant bioaccumulation in marine species and biomagnification along the food chain which would lead to severe consequences also for human health. In this regard, the importance of the approach suggested in the present work is further highlighted by displaying a detailed evaluation of multiple aspects concerning the contaminant characteristics and conditions of the marine-coastal environment.

Besides the slightly negative concentration profile observed for C-2, As concentration in marine biota does not display any significant dependence with the corresponding content in sediments for C-1 and C-3. Marine chemistry of As is indeed characterised by many complex phenomena which cause different biological and chemical transformations, consequently influencing As bioavailability and related toxicity. In marine sediments, As can be mainly present either as arsenate (As(V)) or arsenite (As(III)) depending on the solid matrix layers (i.e. oxidised or reduced, respectively). The occurrence of As release to the water column and the resulting bioaccumulation by marine biota is strictly related to changes of redox conditions (Dang et al., 2014). A further source of As in marine water is represented by the formation of organic forms, such as methylated As, due to aerobic or anaerobic bacteria in sediments as well as phytoplankton and algae. Though methylated forms are generally characterised by lower toxicity than inorganic ones, they can be more efficiently metabolised and accumulated in marine species mostly as arsenobetaine (Duncan et al., 2015; Zhang et al., 2016). Moreover, due to biological excretion and dead materials containing As, further deposit in marine sediments and biodegradation with inorganic As can occur (Kalia & Khambholja, 2015). Therefore, identification of specific As release/accumulation trends between marine sediments and biota can be expected to be challenging. Nonetheless, involvement of different analyses aimed at investigating the physical–chemical and biological conditions of the marine environment could be useful to provide more information about As bioavailability. Also, a statistical approach coupled with monitoring activities is beneficial for the supervision of contaminants distribution and the prediction of potential bioaccumulation increase in the food chain.

## Conclusions

The evidence resulting from the statistical analysis was comprehensively evaluated in order to assess the quality trend of the marine-coastal areas over the whole monitoring period and identify contaminant distribution classes with reference to PTEs content in marine sediments. Further aims consisted in finding possible sources of the contaminants in marine sediments and obtaining an overall scenario about the potential environmental and sanitary risks deriving from the contaminant bioavailability to the marine biota. HCA results identified three main classes along the regional coastline indicating that proper attention should be oriented to different contaminant profiles according to the investigated marine-coastal areas. Correlation analysis suggested the potential common source of different PTEs groups as a function of the HCA classes. Considering As, no prevailing correlation with other PTEs highlighted the need for further in-depth analysis in order to specifically determine its source. However, land use of the areas associated to high As presence also suggested a possible correlation with anthropic sources potentially consisting in industrial and/or agricultural activities. Finally, general observation on the PTEs concentration profiles between sediments and biota usefully indicated that potential contaminant availability to marine species should be considered in each class and for both higher and lower PTEs concentration in the solid matrix. Indeed, comprehension about the factors and mechanisms influencing contaminant transfer between sediments and biota could be further deepened through studies focusing on more complex models to better describe the contaminant concentration trends between matrices. The above-mentioned results support the advantages deriving from the suggested practical approach which is based on multiple statistical analyses and provides integrative information for proper environmental monitoring in the marine-coastal areas. This strategy can represent a fundamental step to develop a comprehensive monitoring model that is useful for environmental quality status assessment and the prevention/control of contaminants distribution. Indeed, as also observed in the present work, informations from several scientific fields are required in order to provide a thorough response for the determination of environmental conditions. Therefore, future studies on this topic should be aimed at developing a multidisciplinary approach connecting scientific support from different research areas, also through the use of statistics.

## Supplementary Information

Below is the link to the electronic supplementary material.

Supplementary file1 (PDF 221 KB)

Supplementary file2 (PDF 80 KB)

## Data Availability

The data of the ARPA Puglia “Operative Monitoring Plan” during 2013, 2014, and 2015 which have been analysed in the current study are available at https://www.arpa.puglia.it/pagina2976_i-ciclo-sessennale-2010-2015.html. The data of the ARPA Puglia “Operative Monitoring Plan” during 2017 which have been analysed in the current study are available at https://www.arpa.puglia.it/pagina2975_ii-ciclo-sessennale-2016-2021.html. The informations on the Puglia Regional Water Protection Plan reported in this study are available at http://www.sit.puglia.it/portal/portale_pianificazione_regionale/Piano%20di%20Tutella%20delle%20Acque/Documenti. Land use data with 8 m resolution and updated to 2011, analysed in the current study, are provided by the Puglia Region and are available at http://www.sit.puglia.it/portal/portale_cartografie_tecniche_tematiche/Cartografie%20Tematiche/UDS.
