# Supplementary material for: Characterising contaminants distribution in marine-coastal sediments through multivariate and nonparametric statistical analyses: a complementary strategy supporting environmental monitoring and control
Source: Environ Monit Assess. 2022 Nov 3;195(1):59. doi: 10.1007/s10661-022-10617-4 (PMC9633496; doi:10.1007/s10661-022-10617-4)
Supplement: Supplementary file 1 — Supplementary file1 (PDF 221 KB) [file 10661_2022_10617_MOESM1_ESM.pdf]

Characterising contaminants distribution in marine-coastal sediments through multivariate and nonparametric statistical analyses: a complementary strategy supporting environmental monitoring and control

Alberto Ferraro[a*], Alessandro Parisi[a], Enrico Barbone[b], Marco Race[c], Matilda Mali[a], Danilo Spasiano[a], Umberto Fratino[a]

Affiliation:

[a] Department of Civil, Environmental, Land, Building Engineering and Chemistry, Polytechnic University of Bari, Via E. Orabona 4, Bari, 70125, Italy

[b] Environmental Protection and Prevention Agency of Puglia Region (ARPA Puglia), Corso Trieste 27, Bari, 70126, Italy

[c] Department of Civil and Mechanical Engineering, University of Cassino and Southern Lazio, Via di Biasio 43, Cassino, 03043, Italy

[*] **Corresponding Author:**

A. Ferraro (alberto.ferraro@poliba.it)

Supplementary material

Table S1. Area code, denomination and HCA class for each monitoring year of the homogeneous marine-coastal SWBs. "NC" indicates the "not classified" marine-coastal SWBs characterized by incomplete set of PTEs concentration data over the whole monitoring period. Red, orange and green colored cells indicate the marine-coastal SWBs of the C-1, C-2 and C-3 classes, respectively. Red, orange and green colored rows indicate the marine-coastal SWBs respectively classified in C-1, C-2 and C-3 classes for all the monitored years.

Area code	Area denomination	HCA class			
		2013	2014	2015	2017
M01	Tremiti Islands	C-3	C-3	C-3	C-3
M02	Chieuti – Fortore mouth	NC	NC	NC	NC
M03	Fortore mouth – Schiapparo mouth	NC	NC	NC	NC
M04	Schiapparo mouth – Capoiale mouth	C-3	C-3	C-3	C-3
M05	Capoiale mouth – Varano mouth	C-1	C-3	C-3	C-3
M06	Varano mouth – Peschici	C-3	C-3	C-3	C-3
M07	Peschici – Vieste	C-1	C-3	C-3	C-3
M08	Vieste – Mattinata	C-1	C-3	C-3	C-3
M09	Mattinata – Manfredonia	C-3	C-3	C-3	C-3
M10	Manfredonia – Cervaro stream	C-3	C-3	C-3	C-3
M11	Cervaro stream – Carapelle mouth	C-3	C-3	C-3	C-3
M12	Carapelle mouth – Aloisa mouth	C-2	C-3	C-3	C-3
M13	Aloisa mouth – Margherita di Savoia	C-1	C-1	C-2	C-3
M14	Margherita di Savoia – Barletta	C-1	C-1	C-1	C-1
M15	Barletta – Bisceglie	C-1	C-1	C-1	C-1
M16	Bisceglie – Molfetta	C-1	C-1	C-1	C-1
M17	Molfetta – Bari	C-2	C-3	C-2	C-2
M18	Bari – San Vito (Polignano)	C-2	C-2	C-2	C-2
M19	San Vito (Polignano) – Monopoli	C-1	C-2	C-2	C-1
M20	Monopoli – Torre Canne	C-2	C-1	C-2	C-3
M21	Torre Canne – North limit of Torre Guaceto protected marine area	C-2	C-3	C-2	C-2
M22	Torre Guaceto protected marine area	C-2	C-3	C-3	C-2
M23	South limit of Torre Guaceto protected marine area – Brindisi	C-3	C-3	C-3	C-3
M24	Brindisi – Cerano	C-2	C-2	C-2	C-2
M25	Cerano – Le Cesine	C-2	C-2	C-3	C-3
M26	Le Cesine – Alimini	C-3	C-3	C-3	C-3
M27	Alimini – Otranto	C-3	C-3	C-3	C-3
M28	Otranto – Santa Maria di Leuca	NC	NC	NC	NC
M29	Santa Maria di Leuca – Torre San Gregorio	NC	NC	NC	NC
M30	Torre San Gregorio – Ugento	NC	NC	NC	NC
M31	Ugento – South limit of Porto Cesareo protected marine area	NC	NC	NC	NC
M32	South limit of Porto Cesareo protected marine area – Torre Colimena	C-3	C-3	C-3	C-3
M33	Torre Colimena – Torre dell'Ovo	C-3	C-3	C-3	C-2
M34	Torre dell'Ovo – Capo San Vito	C-3	C-3	C-3	C-3
M35	Capo San Vito – Punta Rondinella	C-1	C-3	C-3	C-3
M36	Punta Rondinella – Tara River mouth	C-1	C-1	C-1	C-1
M37	Tara River mouth – Chiatona	C-1	C-1	C-3	C-1
M38	Chiatona – Lato mouth	C-1	C-3	C-3	C-1
M39	Lato mouth – Bradano	C-3	C-3	C-3	C-1

Table S2. Kruskal-Wallis statistics for each contaminant tested by considering the related concentrations in the three HCA classes (C-1, C2 and C-3) respective to the monitoring year. Significant differences were observed for $p < 0.05$.

Variable	2013			2014			2015			2017		
	H[a]	DF[b]	p-value	H[a]	DF[b]	p-value	H[a]	DF[b]	p-value	H[a]	DF[b]	p-value
**As**	20.301	2	<0.0001	16.008	2	0.000	18.256	2	0.000	15.657	2	0.000
**Cr**	22.237	2	<0.0001	16.413	2	0.000	11.045	2	0.004	17302	2	0.000
**Ni**	22.600	2	<0.0001	16.211	2	0.000	10.977	2	0.004	17.525	2	0.000
**Pb**	11.346	2	0.003	12.374	2	0.002	11.740	2	0.003	8.753	2	0.013

[a]H: Kruskall-Wallis test statistic

[b]DF: Degree of freedom

Table S3. Friedman statistics for each contaminant tested by considering the related concentrations among the four monitoring years. Significant differences were observed for $p < 0.05$.

Variable	Q[a]	DF[b]	p-value
**As**	22.884	3	<0.0001
**Cr**	20.544	3	0.000
**Ni**	25.850	3	<0.0001
**Pb**	1.073	3	0.784

[a]Q: Friedman test statistic

[b]DF: Degree of freedom

Table S4. Spearman correlation coefficients among contaminants in both marine sediments and biota in relation to each HCA class. The “S” indicates contaminants related to the marine sediments, the “B” indicates contaminants related to the marine biota. Bold values represent significant correlation at $p < 0.05$.

Variable	As_C-1 (B)	Cr_C-1 (B)	Ni_C-1 (B)	Pb_C-1 (B)	As_C-2 (B)	Cr_C-2 (B)	Ni_C-2 (B)	Pb_C-2 (B)	As_C-3 (B)	Cr_C-3 (B)	Ni_C-3 (B)	Pb_C-3 (B)
**As_C-1 (S)**	-0.077	0.174	0.187	0.204								
**Cr_C-1 (S)**	0.089	0.367	**0.396**	0.290								
**Ni_C-1 (S)**	**-0.551**	0.011	0.100	0.174								
**Pb_C-1 (S)**	0.248	**0.462**	0.381	0.378								
**As_C-2 (S)**					-0.201	-0.387	-0.061	-0.236				
**Cr_C-2 (S)**					0.001	-0.405	-0.127	-0.181				
**Ni_C-2 (S)**					-0.191	-0.335	-0.014	0.012				
**Pb_C-2 (S)**					0.028	0.036	0.184	**0.459**				
**As_C-3 (S)**									0.052	-0.023	-0.193	0.112
**Cr_C-3 (S)**									-0.231	**-0.383**	**-0.374**	**-0.337**
**Ni_C-3 (S)**									**-0.274**	**-0.410**	**-0.440**	-0.178
**Pb_C-3 (S)**									-0.110	-0.244	-0.104	-0.094
