# Supplementary material for: Characterising contaminants distribution in marine-coastal sediments through multivariate and nonparametric statistical analyses: a complementary strategy supporting environmental monitoring and control
Source: Environ Monit Assess. 2022 Nov 3;195(1):59. doi: 10.1007/s10661-022-10617-4 (PMC9633496; doi:10.1007/s10661-022-10617-4)
Supplement: Supplementary file 2 — Supplementary file2 (PDF 80 KB) [file 10661_2022_10617_MOESM2_ESM.pdf]

Links to supporting data

The data of the ARPA Puglia "Operative Monitoring Plan" during 2013, 2014 and 2015 which have been analysed in the current study are available at: https://www.arpa.puglia.it/pagina2976_i-ciclo-sessennale-2010-2015.html. The data of the ARPA Puglia "Operative Monitoring Plan" during 2017 which have been analysed in the current study are available at: https://www.arpa.puglia.it/pagina2975_ii-ciclo-sessennale-2016-2021.html. The information on the Puglia Regional Water Protection Plan reported in this study are available at: http://www.sit.puglia.it/portal/portale_pianificazione_regionale/Piano%20di%20Tutella%20delle%20Acque/Documenti. Land use data with 8 m resolution and updated to 2011, analysed in the current study, are provided by the Puglia Region and are available at: http://www.sit.puglia.it/portal/portale_cartografie_tecniche_tematiche/Cartografie%20Tematiche/UDS.
